# T Cells Exacerbate Lyme Borreliosis in TLR2-Deficient Mice

**DOI:** 10.3389/fimmu.2016.00468

**Published:** 2016-11-03

**Authors:** Carrie E. Lasky, Carmela L. Pratt, Kinsey A. Hilliard, John L. Jones, Charles R. Brown

**Affiliations:** ^1^Department of Veterinary Pathobiology, University of Missouri, Columbia, MO, USA

**Keywords:** *Borrelia burgdorferi*, Lyme disease, mouse, toll-like receptor 2, T cells, arthritis

## Abstract

Infection of humans with the spirochete, *Borrelia burgdorferi*, causes Lyme borreliosis and can lead to clinical manifestations such as arthritis, carditis, and neurological conditions. Experimental infection of mice recapitulates many of these symptoms and serves as a model system for the investigation of disease pathogenesis and immunity. Innate immunity is known to drive the development of Lyme arthritis and carditis, but the mechanisms driving this response remain unclear. Innate immune cells recognize *B. burgdorferi* surface lipoproteins primarily *via* toll-like receptor (TLR)2; however, previous work has demonstrated TLR2^−/−^ mice had exacerbated disease and increased bacterial burden. We demonstrate increased CD4 and CD8 T cell infiltrates in *B. burgdorferi*-infected joints and hearts of C3H TLR2^−/−^ mice. *In vivo* depletion of either CD4 or CD8 T cells reduced *Borrelia*-induced joint swelling and lowered tissue spirochete burden, whereas depletion of CD8 T cells alone reduced disease severity scores. Exacerbation of Lyme arthritis correlated with increased production of CXCL9 by synoviocytes, and this was reduced with CD8 T cell depletion. These results demonstrate T cells can exacerbate Lyme disease pathogenesis and prolong disease resolution possibly through dysregulation of inflammatory responses and inhibition of bacterial clearance.

## Introduction

Lyme disease is the most commonly reported vector-borne illness in the United States, with an estimated 300,000 new cases each year (1). It is caused by infection with the spirochete, *Borrelia burgdorferi* (*Bb*), and is transmitted by *Ixodes* ticks (2). Humans infected with *B. burgdorferi* typically develop an expanding circular rash called erythema migrans as an early sign of infection (3). If not treated with antibiotics early during the infection, the spirochetes disseminate and colonize other tissues, potentially resulting in arthritis, carditis, or neurological disease that may be difficult to treat clinically (4). Despite much effort, the mechanisms *B*. *burgdorferi* uses to evade host immune-mediated clearance and persist in tissues resulting in disease pathogenesis are unclear and the focus of many infectious disease researchers.

In mice, innate immunity is considered to be the primary driver of Lyme arthritis and carditis pathogenesis (5, 6). Genetic control of disease resistance or susceptibility was shown to be independent of adaptive immunity (7). Innate phagocytes recognize *B. burgdorferi* primarily through TLR2-mediated recognition of spirochete surface lipoproteins (8). It was hypothesized that TLR2^−/−^ mice would display an attenuated disease phenotype upon infection with *B*. *burgdorferi*. Surprisingly, TLR2^−/−^ mice had increased Lyme arthritis severity in both disease-resistant C57BL/6 mice and -susceptible C3H/HeJ mice (9, 10). This response was attributed to an increased spirochete load in the tissues of TLR2^−/−^ mice. However, increasing the infectious dose in WT mice does not increase arthritis severity, except in BALB/c mice (11). In addition, arthritis susceptibility has been demonstrated to be independent of spirochete loads in joint tissues (11, 12). Following infection of TLR2^−/−^/*scid* double mutant mice, Lyme arthritis severity was returned to WT control levels, suggesting a role for adaptive immune cells in driving the exacerbated disease severity in TLR2^−/−^ mice (13). Subsequent work identified an increased presence of T cells in the joints of *B. burgdorferi*-infected C3H TLR2^−/−^ mice, suggesting that these cells may drive the increased pathology in TLR2^−/−^ mice (14). In addition, it was demonstrated that increased T cell infiltration into mouse joints was correlated with increased production of IFN-induced chemokines (CXCL9 and CXCL10) by synoviocytes. However, the exact role of increased T cell presence in the joints of TLR2^−/−^ mice was not defined.

In the current study, we advanced this line of inquiry by depleting CD4^+^ or CD8^+^ T cells in C3H TLR2^−/−^ mice infected with *B. burgdorferi*. We found that CD8^+^ T cells were increased in the joints of TLR2^−/−^ mice, whereas both CD8^+^ and CD4^+^ T cells were increased in infected hearts. Depletion of both T cell subsets decreased ankle swelling and joint spirochete loads, but only CD8^+^ T cell depletion lowered arthritis or carditis severity scores. These results highlight the complex regulatory mechanisms that drive disease development and suggest CD8^+^ T cells may have an underappreciated role in driving Lyme disease pathology.

## Materials and Methods

### Animals

Female C3H/HeJ mice 4–6 weeks of age were purchased from The Jackson Laboratory (Bar Harbor, ME, USA). C3H TLR2^−/−^ mice at the N6 generation backcross were generously provided by Dr. Linda Bockenstedt (Yale University) and these were fully backcrossed onto the C3H/HeJ background (N10) in our colony. Animals were given sterile food and water *ad libitum* and housed in a specific pathogen-free facility. All works were done in accordance with the Animal Care and Use Committee of the University of Missouri.

### Bacteria and Infections

Frozen stocks of a virulent, passage 8, clonal isolate of *B. burgdorferi* N40 strain were used for all infections. Stocks were added to 7-mL C-BSK-H medium (Sigma–Aldrich, St. Louis, MO, USA) and grown to log phase at 32°C. Spirochetes were enumerated using dark field microscopy and a Petroff–Hausser counting chamber (Hausser Scientific, Horsham, PA, USA). Spirochete dilutions were made in sterile BSK-H medium such that each mouse was inoculated in each hind foot pad with 50-μL medium containing 5 × 10^4^ spirochetes.

### Antibodies and Reagents

Antibodies for use in flow cytometry were obtained from eBioscience (CD45.2-PerCPCy5.5, CD16/CD32, CD3e-PEeFl.610, CD4-APCeFl.780, CD335-APCeFl.780, CD122-PECy7, IFN-γ-PerCPCy5.5, and F4/80-APCeFl.780) and Leinco (CD8-FITC and Ly6g-APC). Depleting antibodies were obtained from Leinco (GK1.5 for anti-CD4-depleting antibody and YTS-169 for anti-CD8-depleting antibody) and graciously donated by Dr. Helen Mullen (University of Missouri) (YTS-156 anti-CD8-depleting antibody). Collagenase/dispase Version 16 was purchased from Roche and resuspended according to manufacturer’s directions. DNaseI DN-25 was purchased from Sigma and stored at −20°C in 2 mg/mL 50% glycerol and 75 mM NaCl.

### *In Vivo* Depletion of T Cell Subsets

C3H TLR2^−/−^ and WT C3H mice were treated with 400-μg CD8-depleting antibody (15) or 300-μg CD4-depleting antibody (16) i.p. 1 day prior to infection. As a negative control, mice were treated with sterile saline i.p. Every 7 days, CD8 or CD4 depletion was maintained by giving an additional i.p. injection of 250 or 100 μg antibody, respectively.

### Cell Isolation for Flow Cytometry

C3H TLR2^−/−^ and C3H WT mice were infected with *B. burgdorferi* and sacrificed at days 14, 21, 28, 42, and 49 postinfection. Hearts were perfused with 1× PBS, removed, and cut into fine pieces. Ankles were harvested from each mouse by removing the toes and carefully cutting through the knee joint, particularly to avoid bone marrow contamination. Excess muscle tissue was trimmed to reduce blood contamination. Ankles and hearts from each mouse were placed in appropriately labeled 15-mL conical tubes containing 5 mL 1× PBS + 4% FBS, 75 μL diluted DNaseI (0.03 mg), and 50 μL stock collagenase/dispase. These were placed on a rocker at room temperature for 1 h before being placed into sterile Petri dishes with 5 mL of additional RPMI supplemented with 10% FBS. Ankle tissue was carefully flayed apart using sterile rat tooth forceps. Cells from joints and hearts were strained through a 70-μm filter (BD Falcon) into a 50-mL conical tube. Cells were spun at 300 *g*, 4°C, 8 min. Supernatant was removed, and cells were washed with 5-mL 1× PBS + 4% FBS three times. Live cells were counted using 3% acetic acid with methylene blue (Stemcell Technologies).

### Flow Cytometric Analysis

For flow cytometry, cells were stained for cell-specific markers. A total of 1 × 10^6^ cells from each sample were placed in a 96-well U-bottom plate and Fc receptors blocked with anti-CD16/CD32 for 15 min at 4°C. Cells were then incubated with cell-specific marker antibodies listed above for 30 min on ice and then washed and fixed in 1% paraformaldehyde for 15 min. Cell analysis was run on a Dako Cyan flow cytometer using Summit V5.0 software. Samples were first gated on live cells, and then doublets were removed. Hematopoietic cells were selected by gating on CD45.2^+^ cells and then specific cell types within that gate were analyzed. For T cells, CD3e^+^ cells were selected and then CD4^+^ or CD8^+^ cell percentages were determined from the total CD3^+^ cell population. Total cell numbers were determined by multiplying the total cells counted in the homogenized tissue × percent CD45.2^+^ cells × percent cell-lineage-specific marker. For T cells, the total cells in the homogenized tissue × percent CD45.2^+^ cells × percent CD3e^+^ cells × percent CD4^+^ or CD8^+^ cells.

### Assessment of Arthritis and Carditis Pathology

Ankle swelling was measured throughout the infection at the thickest craniocaudal portion of the joint using a metric caliper. Arthritis and carditis severity scores were determined, as described previously (17). Zinc–formalin-fixed, paraffin-embedded sections of ankle joints and hearts were stained with hematoxylin and eosin (H&E) and evaluated in a blinded manner on a scale of 0–4 with 0 representing no inflammation and 4 representing severe inflammation in more than half of the section evaluated.

### Determination of *B. burgdorferi* Loads

DNA was extracted from bladders of untreated, CD4-depleted, and CD8-depleted TLR2^−/−^ and WT mice by homogenization in TRIzol as per manufacturer’s instructions. Real-time PCR reactions for *B. burgdorferi flagellin* normalized to copies of mouse *nidogen* within the same sample were performed using TaqMan Universal PCR Master Mix (Applied Biosystems). *Borrelia* loads are expressed as copies of *flagellin* per 1000 copies of *nidogen* as described (18).

### Determination of Antibody Levels

Sera of infected animals were collected at sacrifice by cardiac puncture and *B. burgdorferi*-specific IgM and IgG levels were detected using enzyme-linked immunosorbent assays (ELISAs) on Immulon 2B ELISA plates as described (10).

### Determination of Cytokine Levels in Tissues

Protein was extracted from joint or heart tissue, as described previously (18). Briefly, joint and heart samples were excised and immediately flash frozen in liquid nitrogen. The samples were wrapped in foil and pulverized with a hammer. The resultant powder was resuspended in HBSS-containing 0.2% protease inhibitor mixture (Sigma) and 0.4% Triton X-100. Samples were homogenized using a tissue homogenizer, and particles removed by centrifugation and filtration through a 0.45-μm filter. Samples were brought to 1.5 mL in homogenization buffer. Protein levels were determined using a BCA assay (Pierce), and cytokine levels were determined using a Cytokine Mouse 20-Plex Panel (Life Technologies).

### Statistical Analysis

Statistical analyses were performed using Graphpad Prism software. For single comparisons, an unpaired Student’s *t*-test was performed, and for non-parametric data, we used Mann–Whitney. Multiple comparisons were performed using ANOVA and Tukey *post hoc* test or Dunnett’s test for comparison to a single control (α = 0.05 for all tests).

## Results

### Increased T Cell Infiltration in TLR2^−/−^ Mice

**Figure 1:**
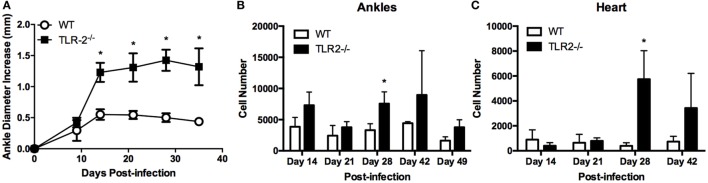
**Arthritis development and T cell infiltrates in TLR2^−/−^ mice**. **(A)** Ankle diameters of WT and TLR2^−/−^ mice were measured weekly following footpad inoculation with 5 × 10^4^ *B. burgdorferi*. At each indicated time point, three mice per strain were sacrificed and numbers of CD3e^+^ T cells were determined from **(B)** ankles or **(C)** hearts by flow cytometry. Data are representative of two separate experiments. Symbols represent means ± SD. *p > 0.01 compared to WT control from same time point.

Intradermal inoculation of *B. burgdorferi* into C3H TLR2^−/−^ mice results in exacerbated arthritis compared with WT C3H and has been correlated with increased numbers of T cells within the joint tissue (9, 14). To investigate this phenomenon further, we infected C3H WT and C3H TLR2^−/−^ mice with *B. burgdorferi* in both rear footpads and followed the development of arthritis. By the second week of infection, the TLR2^−/−^ mice displayed significantly greater ankle swelling than the WT control mice, and this exacerbated response continued past day 35 postinfection (Figure 1A). Mice were sacrificed at various time points, and ankle and heart tissues were processed into single cell suspensions for analysis by flow cytometry. In agreement with a previous report, we found that T cells (CD3^+^ cells) were significantly increased in joint tissue from TLR2^−/−^ mice (Figure 1B). In addition, we also found that T cells were also increased in the inflammatory infiltrates in the hearts of *B. burgdorferi*-infected mice (Figure 1C). Thus, T cells are increased in the inflammatory infiltrates in Lyme arthritis and carditis in TLR2^−/−^ mice.

**Figure 2:**
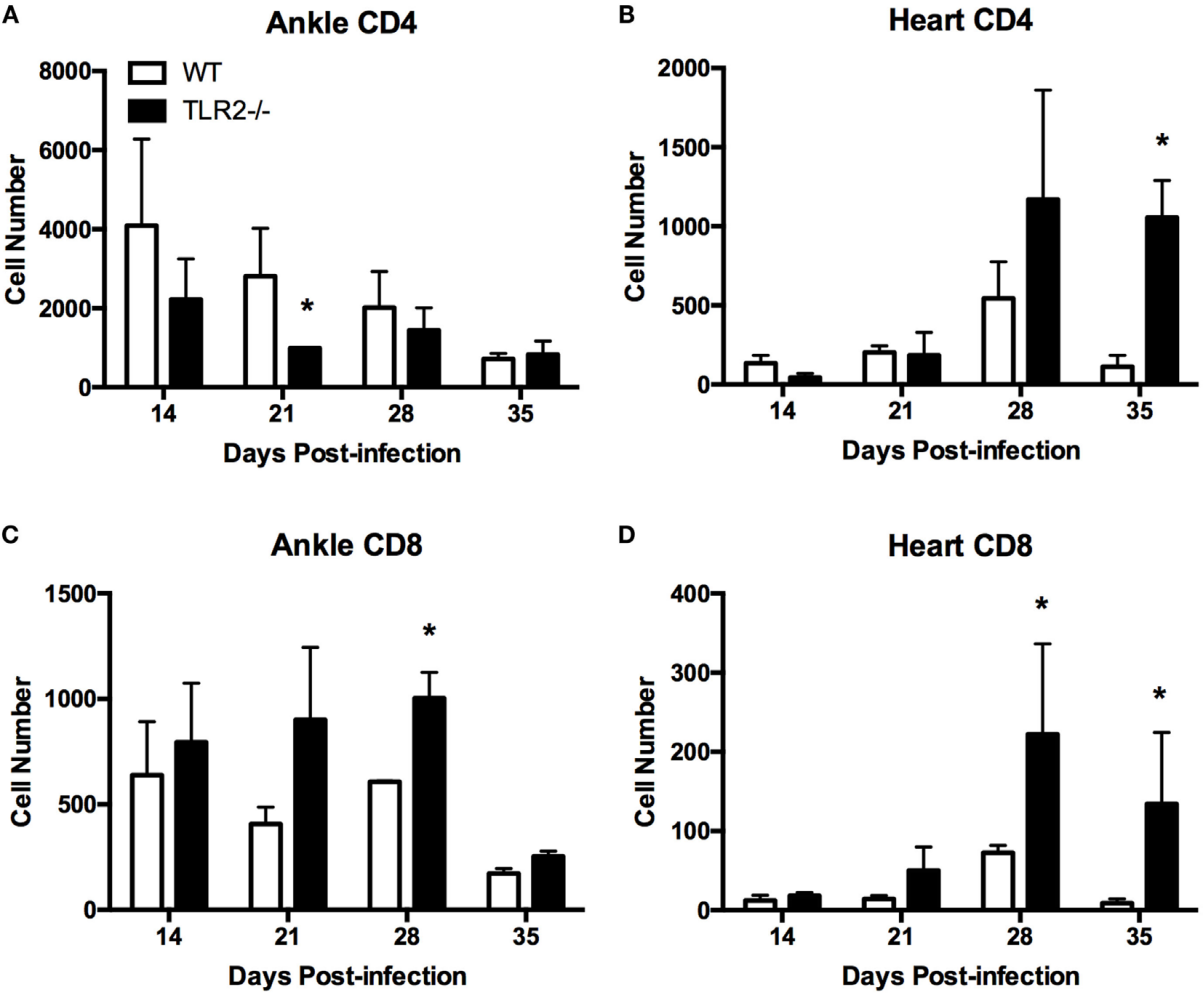
**T cell subsets in infected ankle and heart tissue**. WT and TLR2^−/−^ mice were infected with *B. burgdorferi* and three mice from each strain sacrificed at the indicated time points. T cell subsets in ankle **(A,C)** and heart **(B,D)** tissue were characterized for CD4 **(A,B)**, CD8 **(C,D)** by flow cytometry. Data are representative of two separate experiments. Bars represent means + SD. *p > 0.05 compared to WT control from same time point.

The previous report by Wang et al. (14) did not report the phenotype of the T cells in the increased inflammatory infiltrate in TLR2^−/−^ mice. Therefore, we next investigated the levels of CD4^+^ and CD8^+^ T cell phenotypes in the joints and heart tissue using flow cytometry. CD4^+^ and CD8^+^ T cells were identified from CD45.2^+^ CD3e^+^ T cells. Foxp3^+^ T regulatory cells could not be identified in either joint or heart cellular infiltrates (data not shown). Within both the ankle joint and heart tissue, CD4^+^ T cells made up the majority of the T cells in both WT and TLR2^−/−^ mice (Figure 2). However, whereas CD4^+^ T cells in the joints of TLR2^−/−^ mice were reduced compared to their WT counterparts, in the heart, CD4^+^ cells were increased dramatically near the peak of inflammation (Figures 2A,B). CD8^+^ T cell numbers trended higher at all time points evaluated within the joints and were significantly elevated in the TLR2^−/−^ mice at day 28 postinfection (Figure 2C). In the hearts, CD8^+^ T cells mirrored the increase in CD4^+^ cells and significantly increased near the peak of inflammation (Figure 2D). These results demonstrate that lymphocytes can be differentially recruited into *B. burgdorferi*-infected tissues as has been demonstrated for macrophages and neutrophils (19, 20). In addition, CD8^+^ T cell numbers are increased in the joints of *B. burgdorferi*-infected TLR2^−/−^ mice, while both CD4^+^ and CD8^+^ cells were increased in the infected hearts.

**Figure 3:**
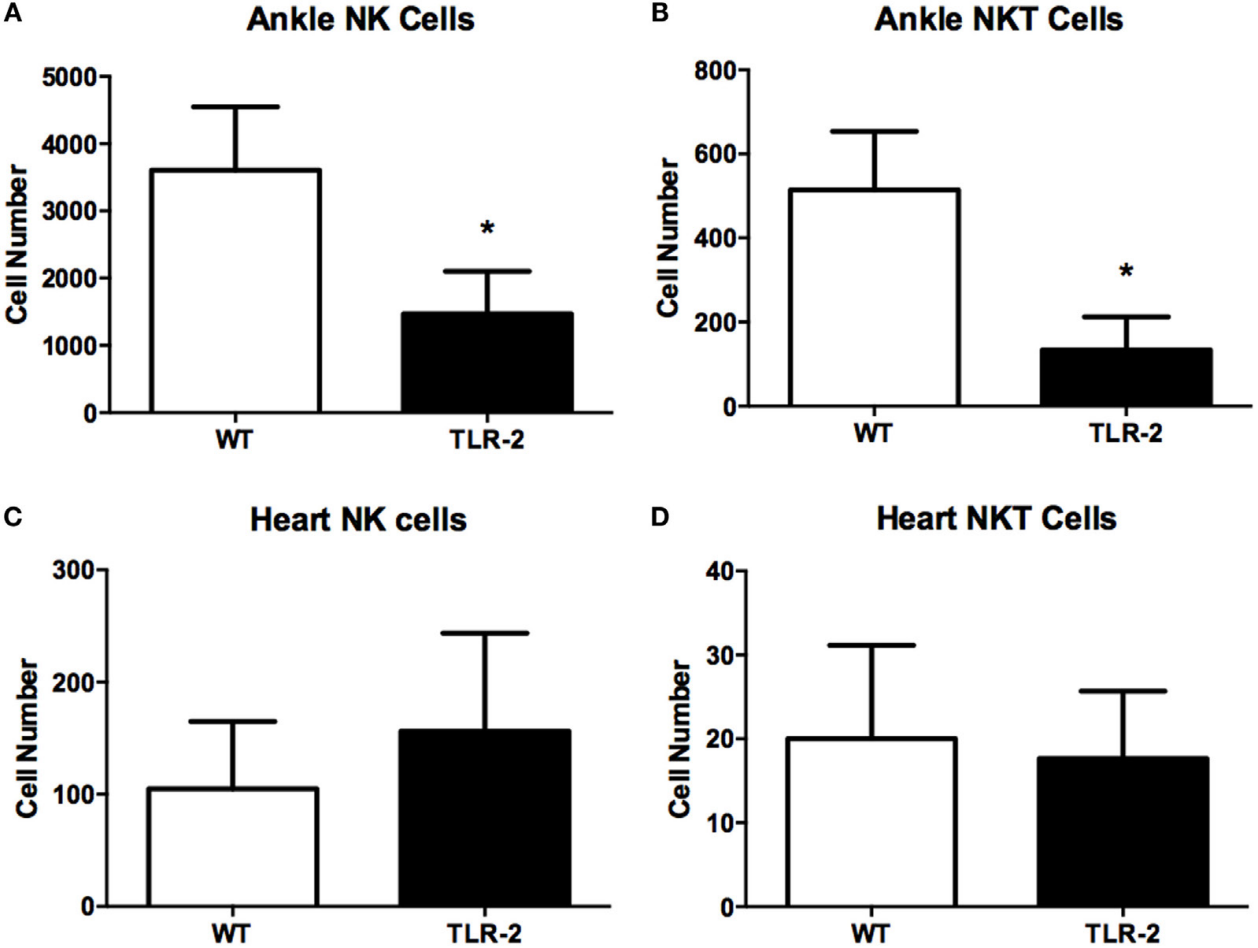
**NK and NKT cells in infected ankle and heart tissue**. WT and TLR2^−/−^ mice were infected with *B. burgdorferi* and three mice from each strain sacrificed at the indicated time points. NK (CD45.2^+^NKp46^+^CD122^+^CD3e^−^) and NKT (CD45.2^+^NKp46^+^CD122^+^CD3e^+^) cells were enumerated in ankle **(A,B)** and heart **(C,D)** tissue at day 21 postinfection. Data are representative of two separate experiments. Bars represent means + SD. *p > 0.05 compared to WT control from same time point.

We also investigated the presence of natural killer T (NKT) cells within the joint or heart tissue of *B. burgdorferi*-infected TLR2^−/−^ mice. NKT cells have been reported to play a role in host defense in both Lyme arthritis and carditis (21, 22). In addition, we also examined the numbers of natural killer (NK) cells in the joints and hearts of TLR2^−/−^ mice, as these cells have been implicated in the exacerbated development of Lyme arthritis in C57BL/6 IL-10^−/−^ mice (23). We therefore looked for both NK (CD45.2^+^NKp46^+^CD122^+^CD3e^−^) and NKT (CD45.2^+^NKp46^+^CD122^+^CD3e^+^) cells in the infected tissues at day 21 postinfection using flow cytometry. Within the infected ankle joints, both NK and NKT cells were significantly decreased in TLR2^−/−^ mice compared to WT mice (Figures 3A,B). Within the infected heart tissue, the numbers of NK cells and NKT cells were quite low and did not differ between WT and TLR2^−/−^ mice (Figures 3C,D). These results suggest that neither NKT nor NK cells are likely responsible for the increased disease severity seen in the TLR2^−/−^ mice.

### *In Vivo* T Cell Subset Depletion in TLR2^−/−^ Mice Reduces Lyme Arthritis to WT Levels

**Figure 4:**
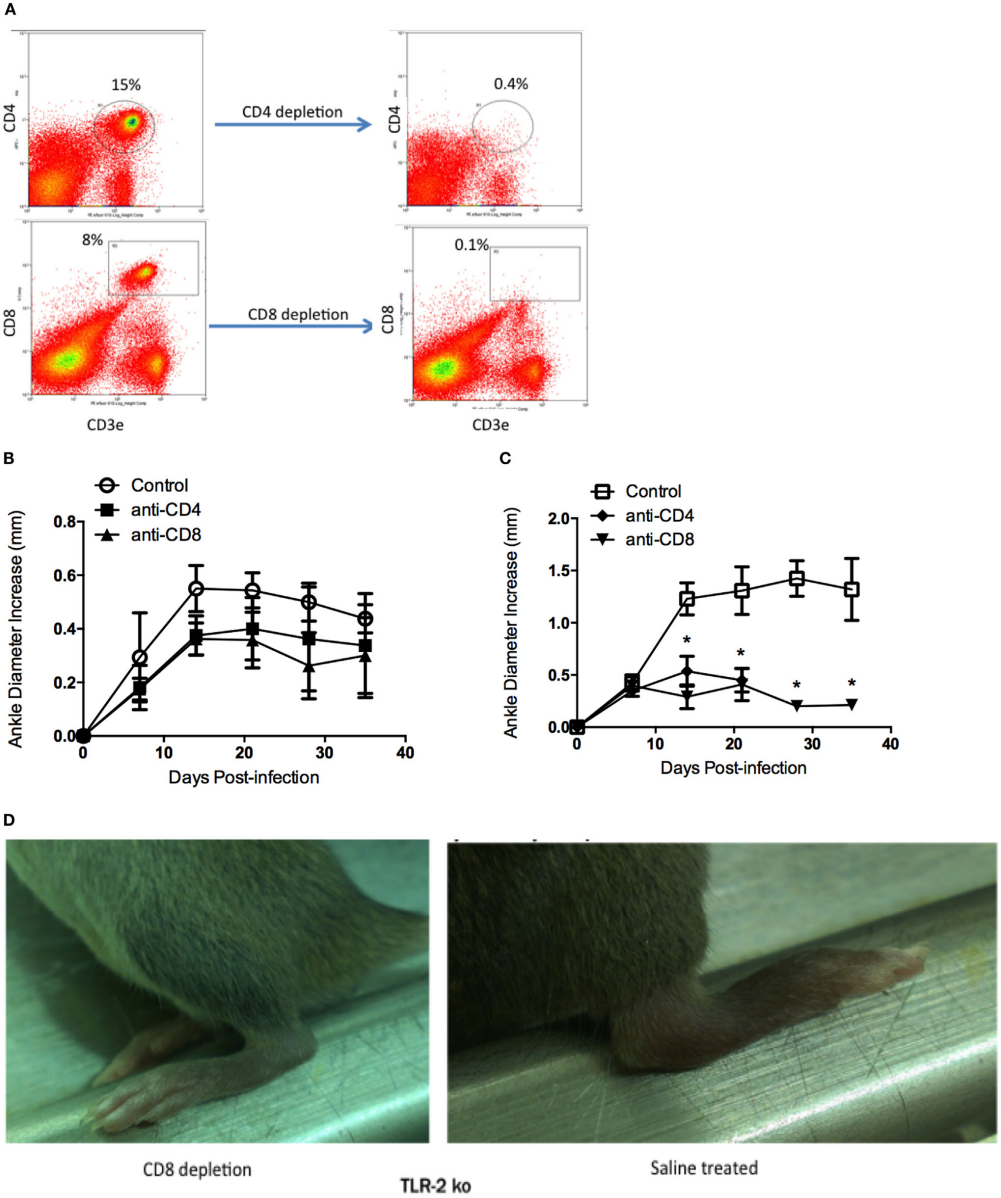
**Lyme arthritis in mice depleted of CD4 or CD8 T cells**. Mice were treated with saline (control), CD4-depleting antibody, or CD8-depleting antibody and infected with *B. burgdorferi*. Flow cytometry plots demonstrate depletion of CD4^+^ and CD8^+^ T cells 6 days posttreatment **(A)**. Arthritis development was monitored over time for WT **(B)** and TLR2^−/−^ **(C)** mice. Representative pictures of TLR2^−/−^ mice depleted of CD8 T cells or saline treated were taken at day 14 postinfection **(D)**. Data are representative of two separate experiments. *n* = 3, symbols represent means ± SD. *p > 0.01 compared to WT control from same time point.

C3H WT and C3H TLR2^−/−^ mice were depleted of either CD4^+^ or CD8^+^ T cells using intraperitoneal injections of either CD4^+^ or CD8^+^ T cell-depleting antibodies. Flow cytometry demonstrated the efficacy of the treatment (Figure 4A). One day after administration of depleting antibodies, mice were infected with 1 × 10^5^ *B. burgdorferi via* footpad inoculation. T cell depletion was maintained by weekly administration of depleting antibody throughout the infection time course. Ankle swelling was monitored every 7 days. WT mice depleted of either CD4^+^ or CD8^+^ T cells had slightly reduced swelling at all time points assessed compared to saline-treated controls, but these differences did not reach statistical significance (Figure 4B). TLR2^−/−^ mice depleted of CD4^+^ or CD8^+^ T cells had significantly reduced ankle swelling compared to untreated controls, and the ankle diameter increase was similar to that typical of WT mice (compare Figures 4B,C). In the experiment shown, the anti-CD4 treated all died between days 21 and 28 postinfection. In a subsequent experiment, the anti-CD4-treated mice had a similar reduction in ankle swelling, but the mice did not die. The reason for this discrepancy is unknown, but in both instances the T cell depletion resulted in a significant decrease in ankle swelling. Figure 4D shows a representative ankle from a TLR2^−/−^ CD8-depleted mouse and a saline-treated TLR2^−/−^ control mouse at day 14 postinfection. The joint swelling in the CD8^+^ T cell-depleted TLR2^−/−^ mouse is dramatically reduced compared to the saline-treated TLR2^−/−^ mouse. A similar reduction was seen in the CD4^+^ T cell-depleted mice (data not shown).

**Figure 5:**
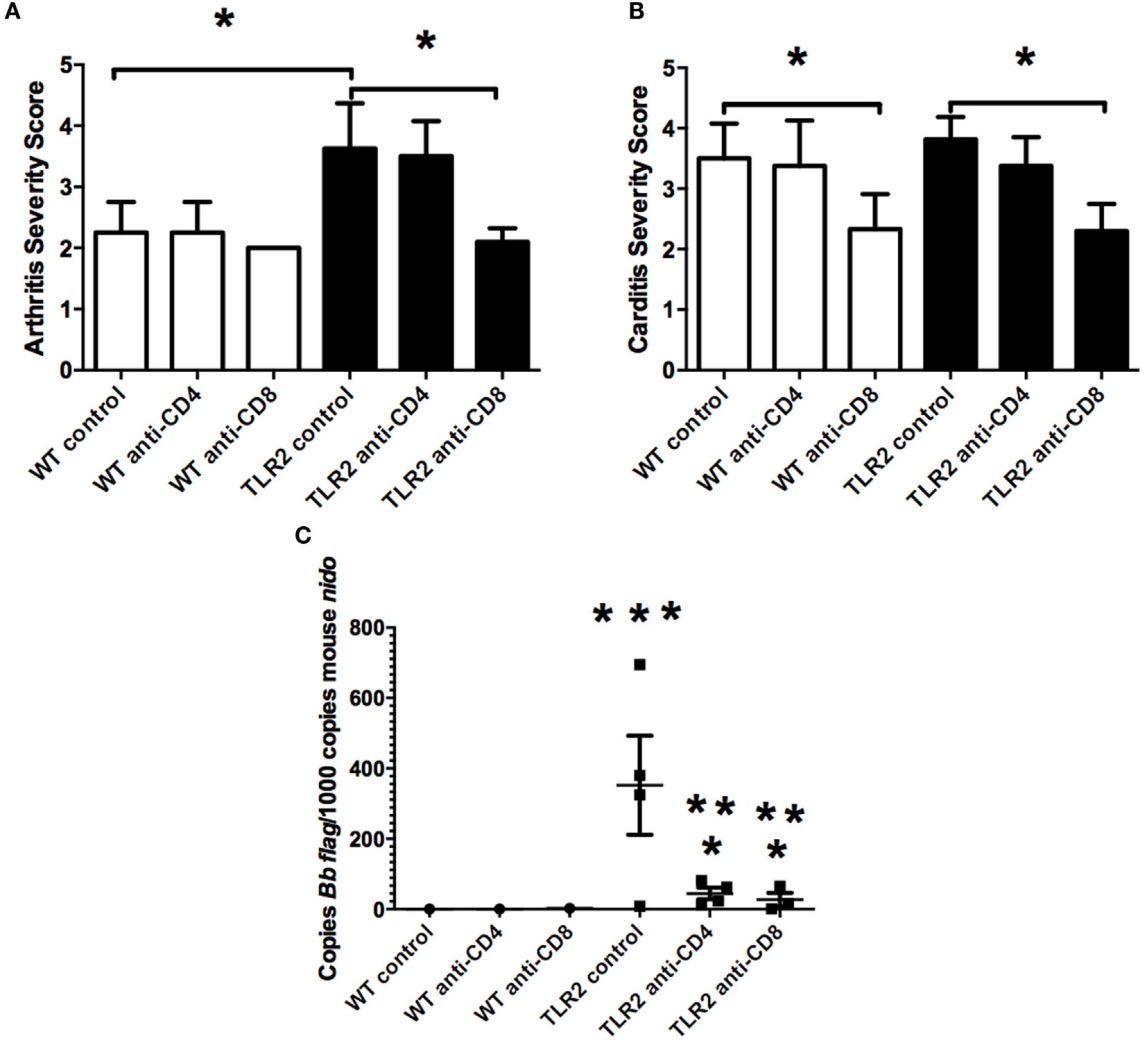
**Arthritis and carditis severity scores**. Mice were infected and treated as in Figure 4. On day 21 postinfection, the mice were sacrificed and ankles **(A)** and hearts **(B)** were processed for histology and scored for lesion severity. Levels of *B. burgdorferi* in tissue were determined by qRT-PCR **(C)**. Data are representative of two separate experiments. *n* = 8, bars represent means ± SD. * vs. TLR2^−/−^ control, ** vs. WT CD4 or CD8-depleted mice, *** vs. WT control, *p* > 0.05.

Sections of both ankle joint and heart tissue from control-treated and T cell-depleted TLR2^−/−^ mice were processed for histology by H&E staining and scored for arthritis and carditis severity (17). As previously reported, arthritis severity scores in TLR2^−/−^ mice were significantly increased over WT control mice at day 21 postinfection (Figure 5A). Depletion of CD4^+^ or CD8^+^ T cell subsets had little effect on arthritis severity scores, with values similar to WT control-treated mice. In contrast, depletion of CD8^+^ T cells, but not CD4^+^ T cells, decreased arthritis severity scores to similar levels as WT mice. CD4^+^ T cell severity scores remained high, similar to the TLR2^−/−^ mice. In the hearts, carditis severity was not increased in the TLR2^−/−^ mice compared to the WT control-treated mice (Figure 5B). Again, as in the joints, depletion of CD4^+^ T cells had no effect on carditis severity scores in either WT or TLR2^−/−^ mice. Depletion of CD8^+^ T cells, however, lowered carditis severity in both the WT and TLR2^−/−^ mice, indicating a role for CD8^+^ T cells in carditis severity. Increased arthritis severity in TLR2^−/−^ mice has been linked to an increase in spirochete burden in joint tissue (10). *B. burgdorferi* loads in tissue were significantly higher in the TLR2^−/−^ control mice than in the WT control mice (Figure 5C). Depletion of T cell subsets had little effect on spirochete levels in WT mice, but significantly lowered *Borrelia* loads in the TLR2^−/−^ mice compared with TLR2^−/−^ control mice. *B. burgdorferi* loads in the CD4^+^ and CD8^+^ T cell-depleted mice were still above levels in T cell-depleted WT mice. Thus, the defect in spirochete clearance in TLR2^−/−^ mice can be mitigated by the removal of either CD4^+^ or CD8^+^ T cells, but does not return them to WT control levels.

**Figure 6:**
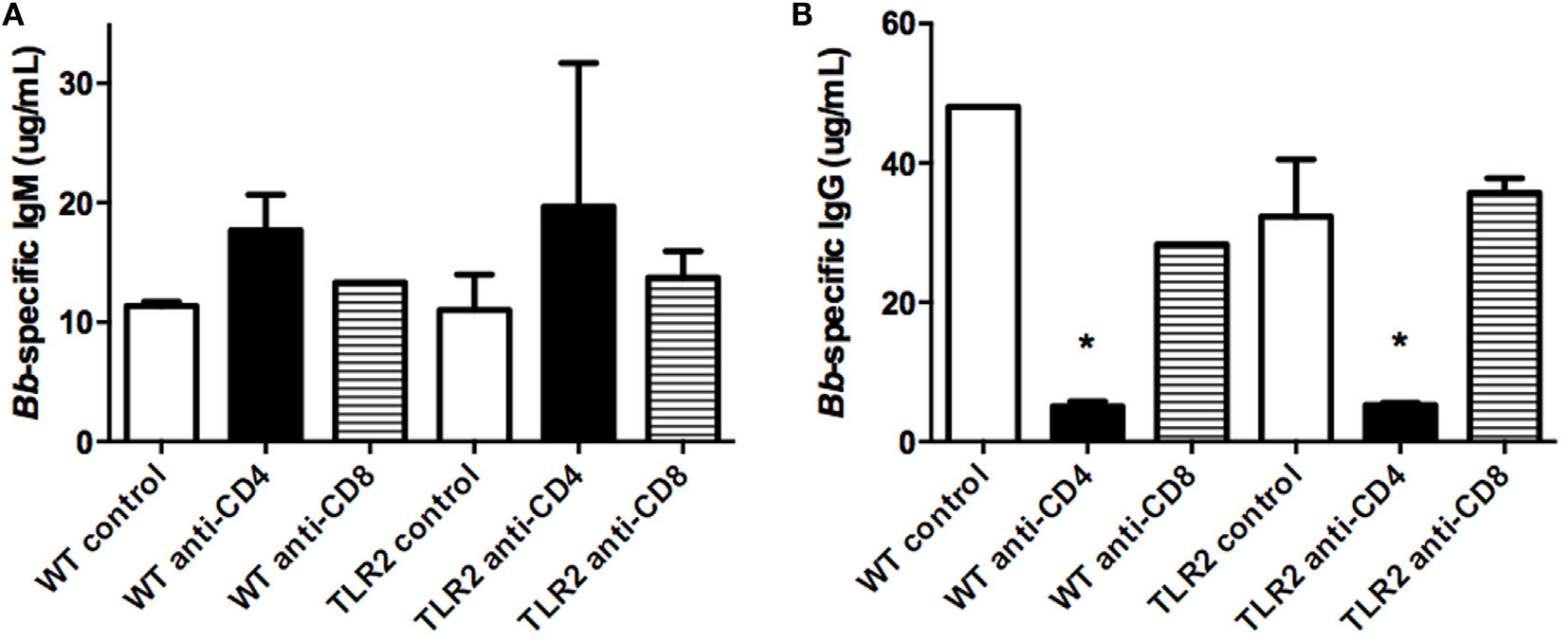
***Borrelia*-specific antibody production**. Mice were infected and treated as in Figure 4. On day 21 postinfection, the mice were sacrificed and assessed for *B. burgdorferi*-specific IgM **(A)** and IgG **(B)** levels in serum. Data are representative of two separate experiments. *n* = 3, bars represent means ± SD. *p > 0.05 compared to mouse strain control.

Production of *Borrelia*-specific antibodies has been reported to be normal in TLR2^−/−^ mice (10). We investigated the effect T cell subset depletion might have on their production. As previously reported, levels of *Borrelia*-specific IgM were not altered in TLR2^−/−^ mice, and there was no effect of T cell depletion on their production (Figure 6A). Similarly, levels of *Borrelia*-specific IgG were no different between WT control and TLR2^−/−^ mice (Figure 6B). However, CD4^+^ T cell depletion significantly decreased *Borrelia*-specific IgG levels in both strains of mice and, thus, was not specific to the TLR2 deficiency, but rather more likely to the lack of T cell help for B cell class switching. CD8^+^ T cell depletion had no effect on IgG levels in either the WT or TLR2^−/−^ mice.

**Figure 7:**
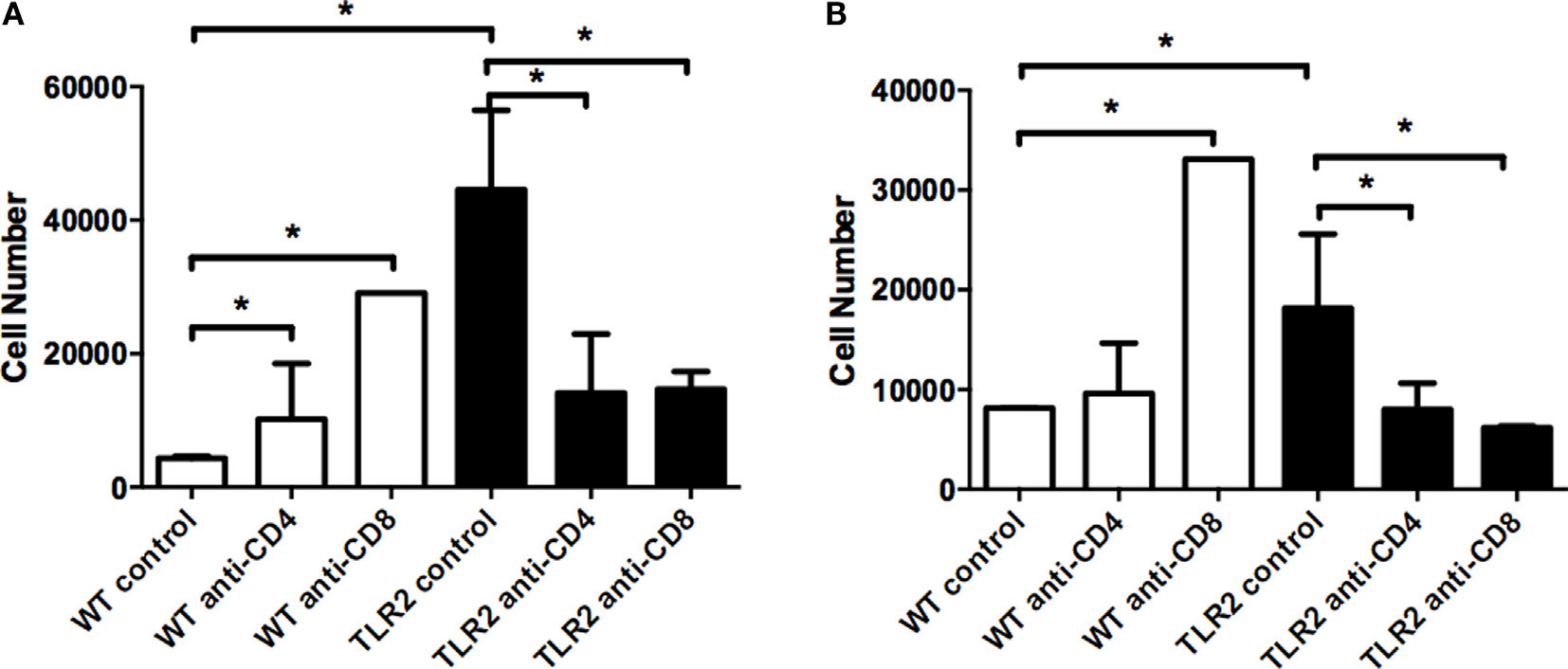
**Neutrophil and macrophage numbers in infected ankle joints**. Mice were infected and treated as in Figure 4. On day 21 postinfection, the mice were sacrificed and neutrophil **(A)** and macrophage **(B)** numbers were assessed from joint tissue. Data are representative of two separate experiments. *n* = 3, bars represent means ± SD. *p > 0.05.

Neutrophils are the predominant inflammatory cell type in the joint in mice during Lyme arthritis, and both neutrophil and macrophage levels increase in the *B. burgdorferi*-infected joints of TLR2^−/−^ mice (14). We therefore investigated the effect of T cell subset depletion on neutrophil and macrophage numbers in the joints of WT and TLR2^−/−^ mice at day 21 postinfection using flow cytometry. Ly6g^hi^ cells, representative of mature neutrophils, were significantly increased in control TLR2^−/−^ mice compared to control WT mice (Figure 7A). Interestingly, following either CD4^+^ or CD8^+^ T cell depletion, neutrophil numbers in joints of WT control mice were significantly increased, although this did not result in an increase in arthritis severity (Figure 5A). In contrast, depletion of either CD4^+^ or CD8^+^ T cells in TLR2^−/−^ mice resulted in a reduction of neutrophil numbers to levels similar to the WT controls. Similar results were seen for macrophage numbers in the *B. burgdorferi*-infected joints, except that CD4^+^ T cell depletion in WT mice did not increase macrophage levels (Figure 7B). These results demonstrate that depletion of T cell subsets in WT mice increases the recruitment of neutrophils and macrophages into the *B. burgdorferi*-infected joint, but does not increase arthritis severity. In contrast, TLR2^−/−^ mice have exacerbated levels of neutrophils and macrophages in the infected joints, and these levels are decreased with T cell depletion, but only CD8^+^ T cell depletion appears to lower arthritis severity.

**Figure 8:**
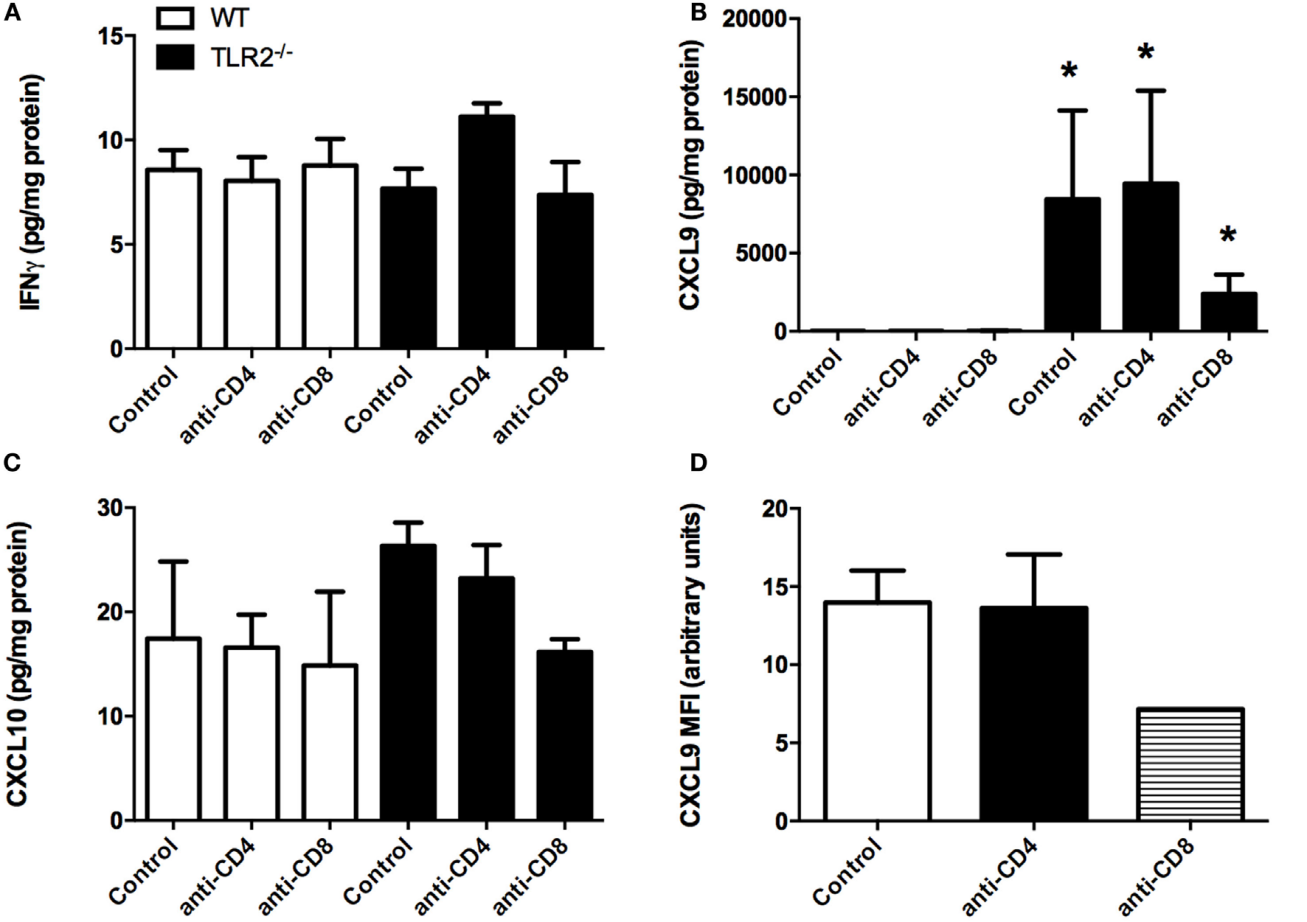
**Cytokine production in joints of infected mice**. Mice were infected and treated as in Figure 4. On day 21 postinfection, the mice were sacrificed and levels of IFNγ **(A)**, CXCL9 **(B)**, and CXCL10 **(C)** were determined from homogenized joint tissue using a Lumina assay. CXCL9 levels within ICAM-1^+^VCAM-1^+^CD14^−^ synoviocytes were assessed from TLR2^−/−^ mice at 14 days postinfection by flow cytometry **(D)**. *n* = 3, data representative of one trial. Bars represent means ± SD. *p > 0.01 compared to WT control.

Bone marrow-derived macrophages from TLR2^−/−^ mice were defective in their production of IL-6, IL-10, and TNFα when stimulated *in vitro* with *B. burgdorferi* recombinant outer surface proteins or sonicated spirochetes (10). In contrast, transcripts from several interferon-inducible chemokine genes were increased in joints of *B. burgdorferi*-infected mice (14). We therefore measured the levels of 20 chemokines and cytokines from the infected joints of control and CD4^+^ or CD8^+^ T cell-depleted WT and TLR2^−/−^ mice at day 21 postinfection using a bead assay system. Most of the cytokines measured were present at low levels, and there were no statistically significant differences between the WT control and TLR2^−/−^ mice (data not shown). Interferon-gamma levels are shown as a representative cytokine for this analysis (Figure 8A). No significant differences were seen between untreated or T cell-depleted WT and TLR2^−/−^ mice. We also looked at an earlier time point, as we have previously shown that many pro-inflammatory mediators peak earlier during Lyme arthritis (18). Analysis of IFN-γ levels in ankle joints at day 7 postinfection yielded no difference between control WT and TLR2^−/−^ mice (data not shown). The only inflammatory mediator that we found to be significantly altered in the joints of the TLR2^−/−^ mice was CXCL9 (Figure 8B). Depletion of CD4^+^ T cells had no effect on the levels of CXCL9, but CD8^+^ T cell depletion dramatically reduced the level of CXCL9 within the joints, although this reduction was not statistically significant. Transcripts of CXCL10 were also reported to increase in joints of TLR2^−/−^ mice infected with *B. burgdorferi* (14), although we found no difference in protein levels in our study (Figure 8C). Synovial cell production of CXCL9- and CXCL10-mediated T cell recruitment and T cell production of mediators that stimulate synovial cell chemokine production set up a synergistic positive feedback loop that has been suggested to drive the enhanced inflammatory response in TLR2-deficient mice (14). We therefore examined the production of CXCL9 from synoviocytes *in vivo* using flow cytometry. Ankle joints from *B. burgdorferi*-infected TLR2^−/−^ mice at day 14 postinfection were processed for flow cytometry. Synoviocytes (ICAM-1^+^V-CAM-1^+^CD14^−^) were found to produce CXCL9 (Figure 8D). Depletion of CD4^+^ T cells had no effect on the production of CXCL9 by these cells; however, CD8^+^ T cell depletion caused about a 50% reduction in CXCL9 production. Due to the low numbers of animals in this experiment, this result was not statistically significant, but along with our previous data, suggests that CD8^+^ T cells are the cells responsible for the increased inflammation and arthritis severity seen in TLR2^−/−^ mice infected with *B. burgdorferi*.

## Discussion

Innate immune cells are the first line of defense against bacterial infection and their responses guide the developing immune response. These cells use an arsenal of pattern recognition receptors to recognize bacterial invaders and stimulate an appropriate response (24). Innate phagocytes recognize extracellular *B. burgdorferi* outer surface lipoproteins primarily *via* TLR1/2 heterodimers (8, 25) and possibly flagellin through TLR5 (26). Other intracellular receptors, such as NOD2, and endosomal TLRs (TLR7, TLR8, and TLR9) may also contribute to the response (27). Therefore, it was surprising when TLR2^−/−^ mice were found to develop exacerbated Lyme arthritis (9, 10). This response was found to require adaptive immune cells (13) and correlated with increased recruitment of T cells into the infected joints (14). Here, we demonstrate that numbers of CD8^+^ T cells, but not CD4^+^ T cells, were increased in the joints of *B. burgdorferi*-infected TLR2^−/−^ mice, but both T cell subsets were increased in the hearts. While depletion of either subset could lower joint swelling and spirochete loads, only CD8^+^ T cell depletion lowered arthritis and carditis severity scores.

Similar to previous publications, we found that TLR2-deficient mice infected with *B. burgdorferi* displayed both increased ankle swelling and elevated numbers of CD3e^+^ T cells within joint tissue at all time points assessed. In infected WT mice, T cells typically make up about 2% of the hematopoietic cell population within the joints. In infected TLR2-deficient mice, this percentage jumped to 10. In the hearts, we found a similar phenomenon; T cell numbers were elevated during carditis. The role of T cells in mediating Lyme disease pathogenesis has been debated for some time. In the mouse model, the balance of CD4^+^ T cell subsets (Th1/Th2) was suggested to mediate Lyme arthritis resistance or susceptibility through the production of IL-4 or IFNγ, respectively (28, 29). Treatment of C3H mice with anti-CD4-depleting antibody increased arthritis severity and treatment with anti-CD8-depleting antibody decreased arthritis severity, suggesting T cell responses could mediate Lyme disease pathogenesis (30). However, a subsequent study in arthritis resistant C57BL/6 mice deficient in CD4^+^ cells showed no difference in the development of Lyme arthritis, but showed a delay in carditis resolution (31). Innate immunity was then shown to be capable of mediating both resistance and susceptibility to Lyme arthritis development (7). The small number of T cells in joints and hearts of *B*. *burgdorferi*-infected mice, however, suggests they might also have a role in shaping the developing immune response. This led to contrasting reports for the role of CD4^+^ T cells in Lyme arthritis and carditis. Adoptive transfer of CD4^+^ T cells into infected B6 RAG^−/−^ mice led to exacerbated arthritis and myocarditis (32), while similar adoptive transfer into B6 TCRα^−/−^ mice resulted in no exacerbated disease and carditis resolution (33). These differences were suggested to be due to the differences in the lymphocyte compartment in the recipient mice and perhaps the induction of autoimmune disease (33). In the current study, we used serodepletion to remove CD4^+^ or CD8^+^ T cell subsets from naive WT or TLR2^−/−^ mice. We found that depletion of CD4^+^ or CD8^+^ T cells had no effect on arthritis development in C3H mice, although depletion of CD8^+^ cells reduced carditis severity at 3 weeks postinfection. These results are similar to those of Fikrig et al. (31) and contrasting with those of Keane-Myers and Nickell (30). The reasons for these differences are not clear, but most likely are due to differences in antibody treatments and efficiency of cell subset depletions. A role for CD8^+^ T cells in driving exacerbated disease in the TLR2^−/−^ mice, however, has not been previously reported and may represent a new target for therapy as CD8^+^ T cells specific for *Borrelia* antigens have been reported in blood of patients with Lyme arthritis (34). We also looked the presence of T regulatory cells in the joints and hearts of WT and TLR2^−/−^ mice, since these have been suggested to play a role in Lyme disease pathogenesis (35). T regulatory cells could not be found in either joint or heart tissue, and in the current, we did not look for γδT cells.

Natural killer cells are induced in mice following infection with *B. burgdorferi*, but their depletion had little effect on arthritis pathogenesis (36). They have been suggested to contribute to the increased inflammation seen in B6 IL-10^−/−^ mice *via* their production of IFNγ (23). Prolonged NK cell activity has also been correlated with continued inflammation following antibiotic therapy in Lyme patients (37). We found decreased NK cell numbers in the joints of TLR2^−/−^ mice and no change in heart tissue. Since there was no change in joint IFNγ levels in the TLR2^−/−^ mice, it is unlikely the NK cells play a major role in the TLR2^−/−^ mice. We found similar results for NKT cells. Low numbers of NKT cells were identified within infected joint tissue and almost none were found within heart tissue. NKT cells recognize bacterial glycolipids presented by CD1d (38), and infection of mice deficient in CD1 with *B. burgdorferi* led to impaired host defense, increased arthritis severity, and impaired spirochete clearance (39). NKT cells specifically recognize diacylglycerol antigens from *B. burgdorferi* (40). They limit spirochete dissemination by killing blood-borne spirochetes in the liver (41) and act as a cytotoxic barrier to prevent spirochete entry into the joint space (42). Deletion of NKT cells resulted in more severe and prolonged arthritis and reduced ability to clear spirochetes from tissues (22). It is tempting to speculate that the reduced numbers of NKT cells in the infected joints of TLR2^−/−^ mice might be responsible for the increased arthritis severity and spirochete loads in this tissue. However, how depletion of CD4^+^ or CD8^+^ cells, both of which are expressed on some NKT cell subsets, could reduce *B. burgdorferi* tissue loads and increase arthritis severity in the TLR2^−/−^ mice is unclear. More work is needed in this area.

Both C57BL/6- and C3H TLR2-deficient mice infected with *B. burgdorferi* display significantly worse arthritis and carditis (14, 43), and we have shown this is mediated primarily by increased recruitment of CD8^+^ T cells into the infected joint. An increase in T cell numbers in the joints was associated with increased transcript levels of CXCL9 and CXCL10, giving a potential mechanism for the increased T cell recruitment (14). It was suggested that the enhanced arthritis of TLR2^−/−^ mice was due to unregulated local chemokine production by synoviocytes. We measured chemokine production directly from infected joints and found CXCL9 was significantly unregulated in the joints of *B. burgdorferi*-infected TLR2-deficient mice. Protein levels of CXCL10 were only slightly elevated and at much lower levels than CXCL9. In the absence of CD8^+^ T cells, CXCL9 levels were dramatically reduced, suggesting that CD8^+^ T cells activate synoviocytes to produce CXCL9, thereby recruiting additional T cells into the joint and driving increased inflammation in the TLR2^−/−^ mice. CXCL9 production can be induced by either type I or type II IFN (44). Production of type I IFN has been linked to arthritis development in *B. burgdorferi*-infected mice, and its production has been shown to be independent of TLR2 signaling (45). We measured levels of type II IFN (IFNγ) from the joints of *B. burgdorferi*-infected mice and found they were similar between WT and TLR2^−/−^ mice. Thus, the mechanism responsible for increased CXCL9 expression in the joints of TLR2^−/−^ mice is still unclear and is currently being investigated.

## Author Contributions

CL and CB designed the experiments, analyzed the data, and wrote the manuscript. CL, CP, KH, and JJ performed the experiments.

## Conflict of Interest Statement

The authors declare that the research was conducted in the absence of any commercial or financial relationships that could be construed as a potential conflict of interest.
